# 3rd Thymus Workshop: Rolduc 1991

**DOI:** 10.1155/1992/83191

**Published:** 1992

**Authors:** Mary A. Ritter


					Developmental Immunology, 1992, Vol. 2, p. 67
Reprints available directly from the publisher
Photocopying permitted by license only
(C) 1992 Harwood Academic Publishers GmbH
Printed in the United Kingdom
3rd Thymus Workshop"
Rolduc 1991
Long before the European Council of Ministers put Maastricht on the map, scientists researching into the
mysteries of the thymus had discovered and enjoyed the excellent secluded facilities provided by the
Centrum Rolduc in the 12th century monastery nearby. The 3rd Thymus Workshop was attended by
approximately 100 participants who gathered together for 3 days of informal but intensive presentations
and discussions of current research data.
The meeting opened with a guest lecture from Dr. Mike Owen (London) who spoke on the "Regulation
of gene expression during T cell differentiation and activation"; a topic that admirably set the scene for
the Workshop sessions that followed, since an understanding of gene regulation should ultimately
provide the meeting point for the many and varied current areas of thymus research. Such areas include
the sequential steps through which T cells pass during their intrathymic development, the way in which
these steps are controlled via signals provided by the stromal cells of the thymic microenvironment (e.g.
epithelial cells, dendritic cells and macrophages), and the perturbation of these processes in disease
states.
Contributors to these sessions were encouraged to submit manuscripts, based on their presentation, for
publication in Developmental Immunology, subject to normal reviewing procedures. It is these papers
that are now collected together in this special 'Rolduc' volume. Although it is difficult to reproduce in the
written word the highly charged atmosphere of a stimulating scientific meeting, we hope that this
volume will provide the reader with a more detailed presentation of the research work discussed while at
the same time giving a little of the atmosphere enjoyed by the participants.
Mary A. Ritter
67

				

